# A case-based review of urachal carcinoma with mixed small cell neuroendocrine and adenocarcinoma components, with one new case report and analysis of nine published cases

**DOI:** 10.3389/fendo.2025.1676835

**Published:** 2025-11-13

**Authors:** Junhao Chu, Jiwei Zhai, Qimei Ma, Zhihui Zhang, Yanhua Yuan, Huisheng Yuan, Chunxiao Wei, Muwen Wang

**Affiliations:** 1 Department of Urology, Shandong Provincial Hospital Affiliated to Shandong First Medical University, Jinan, China; 2 Central Sterile Supply Department, Shandong Provincial Hospital Affiliated to Shandong First Medical University, Jinan, China; 3 Outpatient Department, Shandong Provincial Hospital Affiliated to Shandong First Medical University, Jinan, China; 4 Department of Andrology, The Seventh Affiliated Hospital, Sun Yat-sen University, Shenzhen, China

**Keywords:** urachal carcinoma, neuroendocrine carcinoma, small cell carcinoma, therapy, case report

## Abstract

**Introduction:**

Urachal carcinoma (UrC) is an uncommon malignant neoplasm arising from urachal remnants and represents only 0.01%–0.7% of bladder cancers. Adenocarcinoma—usually of the intestinal type—accounts for over 80% of cases, whereas neuroendocrine carcinoma (NEC) is exceedingly rare. Fewer than ten cases of urachal NEC have been documented in the English-language literature, most diagnosed at advanced stages with poor outcomes. We report an additional case and review published data to enhance clinical recognition and management of this ultra-rare tumor.

**Case presentation:**

A 43-year-old woman presented to Shandong Provincial Hospital, Shandong First Medical University, after a urachal midline mass was incidentally detected on routine health examination. Preoperative tests showed a CEA level of 7.28 ng/mL. CTU revealed a 3.9 × 2.7 × 2.4 cm cystic–solid lesion at the anterior bladder wall, suspicious for urachal malignancy. Cystoscopic biopsy confirmed small-cell NEC. The patient underwent laparoscopic urachal resection with umbilicus preservation, extended partial cystectomy, and bilateral pelvic lymphadenectomy. Postoperative pathology showed a mixed urachal carcinoma composed of ~80% small-cell NEC and ~20% adenocarcinoma, forming a 4.5 × 3 × 1.5 cm cystic–solid mass. Margins and lymph nodes were negative. Immunohistochemical analysis showed a high Ki-67 labeling index (80%) and positive staining for synaptophysin (Syn), chromogranin A (CgA), insulinoma-associated protein 1 (INSM1), cytokeratin 20 (CK20), and mutant-pattern p53. Retinoblastoma protein (RB) and GATA-3 were negative. The patient received four cycles of adjuvant etoposide–cisplatin (EP) chemotherapy. Surveillance with tumor markers and whole-abdominal CT every three months showed no evidence of recurrence at the 8-month follow-up.

**Conclusion:**

Urachal NEC with mixed small-cell and adenocarcinoma components is an exceptionally rare and highly aggressive malignancy lacking standardized diagnostic or therapeutic guidelines. Complete surgical excision with negative margins remains the mainstay of treatment, while adjuvant regimens are typically adapted from small-cell carcinoma protocols of the lung or urinary tract. We report a case managed with umbilicus-sparing urachectomy and extended partial cystectomy followed by EP chemotherapy, together with a review of nine previously published cases. These findings provide literature-based evidence to guide individualized management and inform future multidisciplinary research.

## 1 Introduction

Urachal carcinoma (UrC) is a rare but aggressive malignancy arising from the urinary tract, representing less than 1% of all bladder cancer cases (1, 2). The first description of UrC was provided by Hue and Jacquin in 1864 (3). Subsequent studies have demonstrated that approximately 90% of UrCs are histologically classified as adenocarcinomas (4). In contrast, non-adenocarcinoma subtypes are exceedingly rare, comprising only around 8% of cases. Among these, urothelial carcinoma is most prevalent, followed by sarcomas, squamous cell carcinomas, and neuroendocrine carcinoma (NEC) (5). Among these, urachal NEC is exceptionally rare, with only nine confirmed cases documented in the English-language literature to date. UrC is associated with a poor prognosis, typically diagnosed between the ages of 52 and 59, with a clear male predominance (6). Early-stage UrC is often asymptomatic, and diagnosis typically occurs at an advanced stage owing to local invasion or distant metastasis. Gross hematuria is the most frequently reported symptom, occurring in approximately 90% of cases. Other manifestations include lower abdominal pain, recurrent urinary tract infections, and palpable masses in the suprapubic region (1) (4). The most commonly involved metastatic sites include the lungs, bones, peritoneum, liver, and pelvic lymph nodes (7). Given its extreme rarity, no standardized treatment guidelines for UrC have been established to date. For localized lesions, surgical resection remains the cornerstone of treatment, typically involving en bloc removal of the urachus and umbilicus, partial or radical cystectomy, and bilateral pelvic lymphadenectomy (8).

We report a rare case of urachal NEC exhibiting mixed histological features of adenocarcinoma and small cell neuroendocrine carcinoma. To the best of our knowledge, this case constitutes the tenth formally published instance of urachal NEC worldwide. The patient underwent laparoscopic urachectomy with preservation of the umbilicus, extended partial cystectomy, and bilateral obturator lymphadenectomy, followed by four cycles of adjuvant etoposide-cisplatin (EP) chemotherapy. At the 8-month follow-up, the patient demonstrated good postoperative recovery with no evidence of disease recurrence. However, owing to its exceptional rarity and the frequent presentation at advanced stages, no standardized diagnostic or therapeutic guidelines have been established to date. This study delineates the comprehensive diagnostic and therapeutic course of the present case, supplemented by longitudinal follow-up, and includes a systematic review of previously reported cases. By integrating this case with the literature, we summarize the clinical presentation, pathological characteristics, diagnostic considerations, and treatment strategies pertinent to this rare entity. We aim to provide conceptual context and practical guidance for the clinical management of this exceptionally uncommon malignancy.

## 2 Case presentation

### 2.1 Preoperative condition

**Figure 1:**
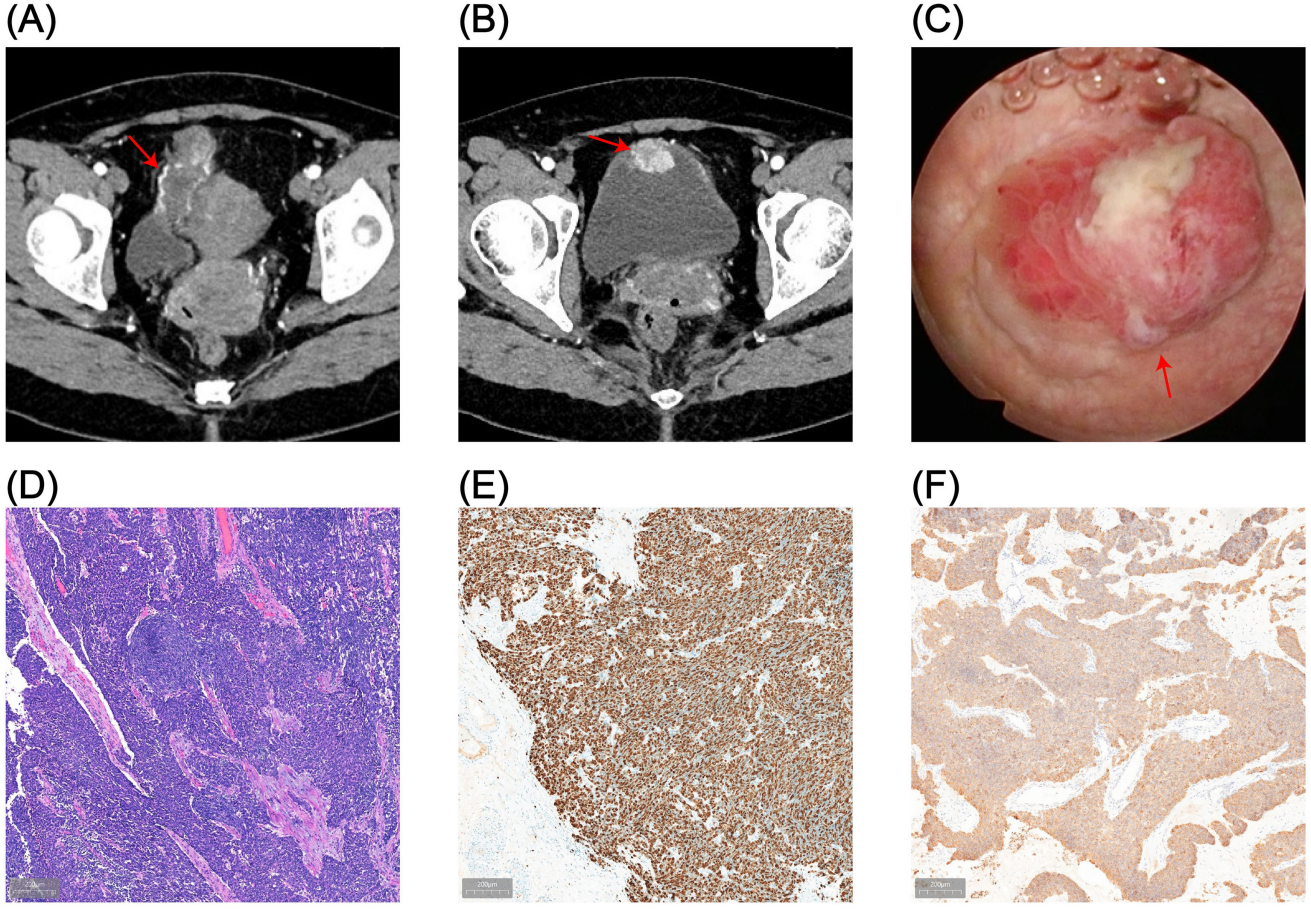
Preoperative imaging and pathological findings. **(A, B)** CTU reveals a cystic-solid mass (3.9 × 2.7 cm) located at the anterior bladder wall, demonstrating transmural invasion and marked heterogeneous enhancement. **(C)** Cystoscopic imaging shows full-thickness infiltration of the anterior bladder wall by the tumor. **(D)** Hematoxylin and eosin (HE) staining of cystoscopic biopsy confirms urachal neuroendocrine carcinoma (NEC) with small cell morphology. **(E, F)** Immunohistochemical staining shows a Ki-67 proliferation index of approximately 80% **(E)** and positive expression of synaptophysin (Syn) **(F)**.

A 43-year-old female patient was admitted to the Department of Urology, Provincial Hospital Affiliated with Shandong First Medical University, on December 12, 2024, following the incidental detection of a urachal mass during a routine health check-up. Laboratory tests revealed an elevated carcinoembryonic antigen (CEA) level of 7.28 ng/mL (reference range: 0–5 ng/mL), and urinalysis indicated gross hematuria with 126.5 red blood cells per high-power field (HPF) (normal ≤3 HPF). Computed tomography urography (CTU) identified a cystic-solid lesion measuring approximately 3.9 × 2.7 × 2.4 cm at the anterior bladder wall, demonstrating significant heterogeneous enhancement, consistent with a suspected urachal malignancy (**Figures 1A, B**). Preoperative cystoscopic biopsy confirmed urachal neuroendocrine carcinoma, predominantly of the small cell morphological subtype. Immunohistochemical analysis demonstrated high Ki-67 proliferative index (80%) and positive expression of synaptophysin (Syn), chromogranin A (CgA), and insulinoma-associated protein 1 (INSM1), along with loss of retinoblastoma (RB) protein expression (**Figures 1C–F**). Based on histopathological and radiographic findings, a preoperative diagnosis of urachal neuroendocrine carcinoma (Mayo stage II) was established.

### 2.2 Surgical procedure and postoperative pathology

**Figure 2:**
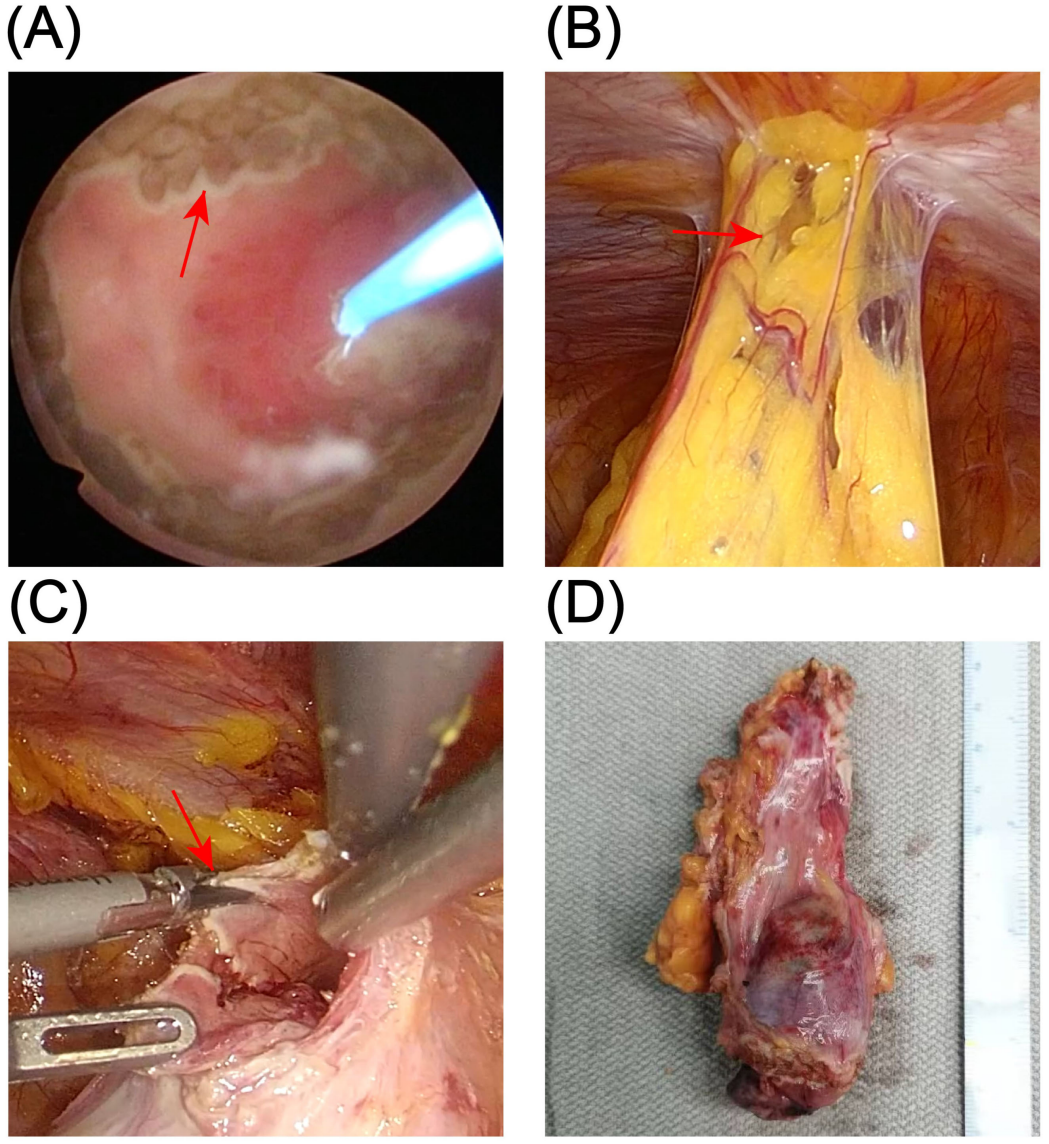
Surgical procedure. **(A)** Circumferential marking of the resection margin with a 1470-nm diode laser under cystoscopic guidance (red arrows). **(B)** Laparoscopic exploration showing that the urachus terminates approximately 5 cm inferior to the umbilicus, without extension to the umbilicus (red arrows). **(C)** Laparoscopic en bloc resection of the tumor along a plane approximately 1.5 cm beyond the laser-marked margin (red arrows). **(D)** Gross appearance of the resected specimen.

**Figure 3:**
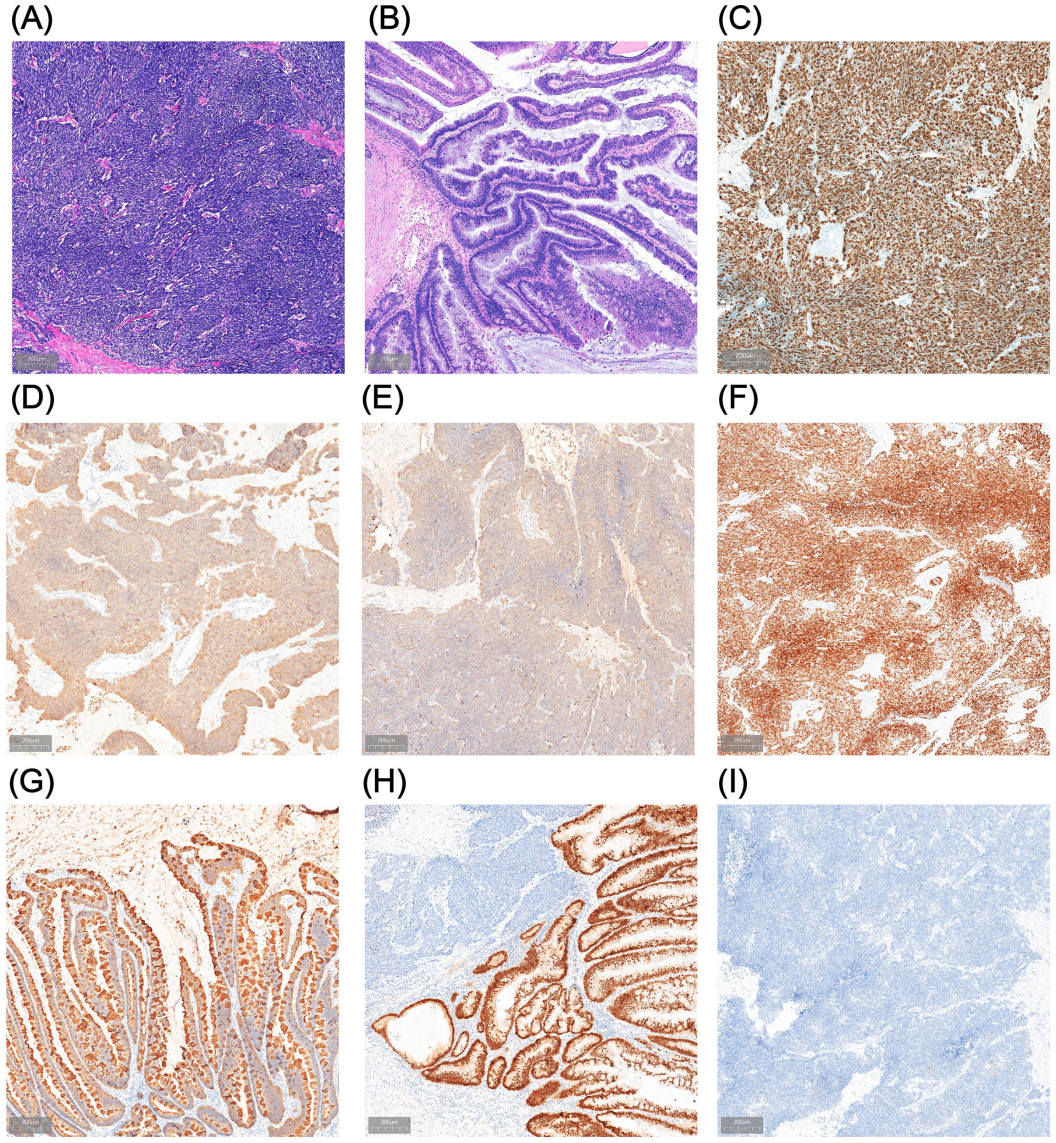
Postoperative pathological findings. **(A, B)** Hematoxylin and eosin (HE) staining of the resection specimen shows urachal neuroendocrine carcinoma (NEC) with small-cell morphology **(A)** admixed with an adenocarcinoma component **(B)**. **(C–I)** Immunohistochemical staining demonstrates a Ki-67 proliferation index of approximately 80% **(C)** and positive expression of synaptophysin (Syn) **(D)**, chromogranin A (CgA) **(E)**, insulinoma-associated protein 1 (INSM1) **(F)**, cytokeratin 20 (CK20) **(G)**, and CDX2 **(H)**, with negative GATA-3 **(I)**.

Considering the patient’s young age, absence of significant lymphadenopathy on preoperative imaging, and strong preference for bladder and umbilical preservation, a multidisciplinary team decided—following informed consent—to perform laparoscopic urachal resection with umbilicus preservation, extended partial cystectomy, and bilateral pelvic lymphadenectomy. Following comprehensive preoperative preparation, the patient underwent surgery on December 17, 2024. To achieve clear demarcation and negative margins for the laparoscopic partial cystectomy, the patient was placed in the lithotomy position under general anesthesia and a cystoscope was introduced. A 1470-nm diode (semiconductor) laser was used (cutting power 100 W, coagulation power 30 W) to circumferentially mark the intended margin approximately 2 cm beyond the tumor edge within the bladder lumen, followed by stepwise vaporization down to the muscularis propria to delineate the planned resection field. The scope was then withdrawn, and a 20-Fr three-way Foley catheter was left in place (**Figure 2A**). The patient was then repositioned to the supine position for laparoscopic exploration. Intraoperatively, the urachus was found to terminate approximately 5 cm below the umbilicus, with no direct extension into the umbilical region (**Figure 2B**). The proximal urachus and its adhesions to the abdominal wall and omentum were resected. Intraoperative frozen section analysis confirmed negative surgical margins. Laparoscopic urachal resection with umbilical preservation, extended partial cystectomy, and bilateral pelvic lymphadenectomy were subsequently completed (**Figures 2C, D**). Postoperative histopathological examination confirmed a mixed urachal carcinoma, predominantly composed of small cell neuroendocrine carcinoma (~80%) (**Figure 3A**) and adenocarcinoma (~20%) (**Figure 3B**) components. The tumor presented as a cystic-solid mass measuring approximately 4.5 × 3 × 1.5 cm. No tumor involvement was detected at the surgical margins or in bilateral pelvic lymph nodes. Immunohistochemical analysis demonstrated a high Ki-67 labeling index (80%) and positive staining for synaptophysin (Syn), chromogranin A (CgA), insulinoma-associated protein 1 (INSM1), cytokeratin 20 (CK20), caudal-type homeobox 2 (CDX2), and mutant-pattern p53, whereas retinoblastoma protein (RB) and GATA-binding protein 3 (GATA-3) were negative (**Figures 3C–I**). Based on the final histopathological findings, the patient was diagnosed with mixed small cell neuroendocrine carcinoma of the urachus (Mayo stage II). The urinary catheter was successfully removed two weeks postoperatively, after which the patient voided spontaneously with an unobstructed stream and remained in good clinical condition.

### 2.3 Postoperative management and follow-up

**Figure 4:**
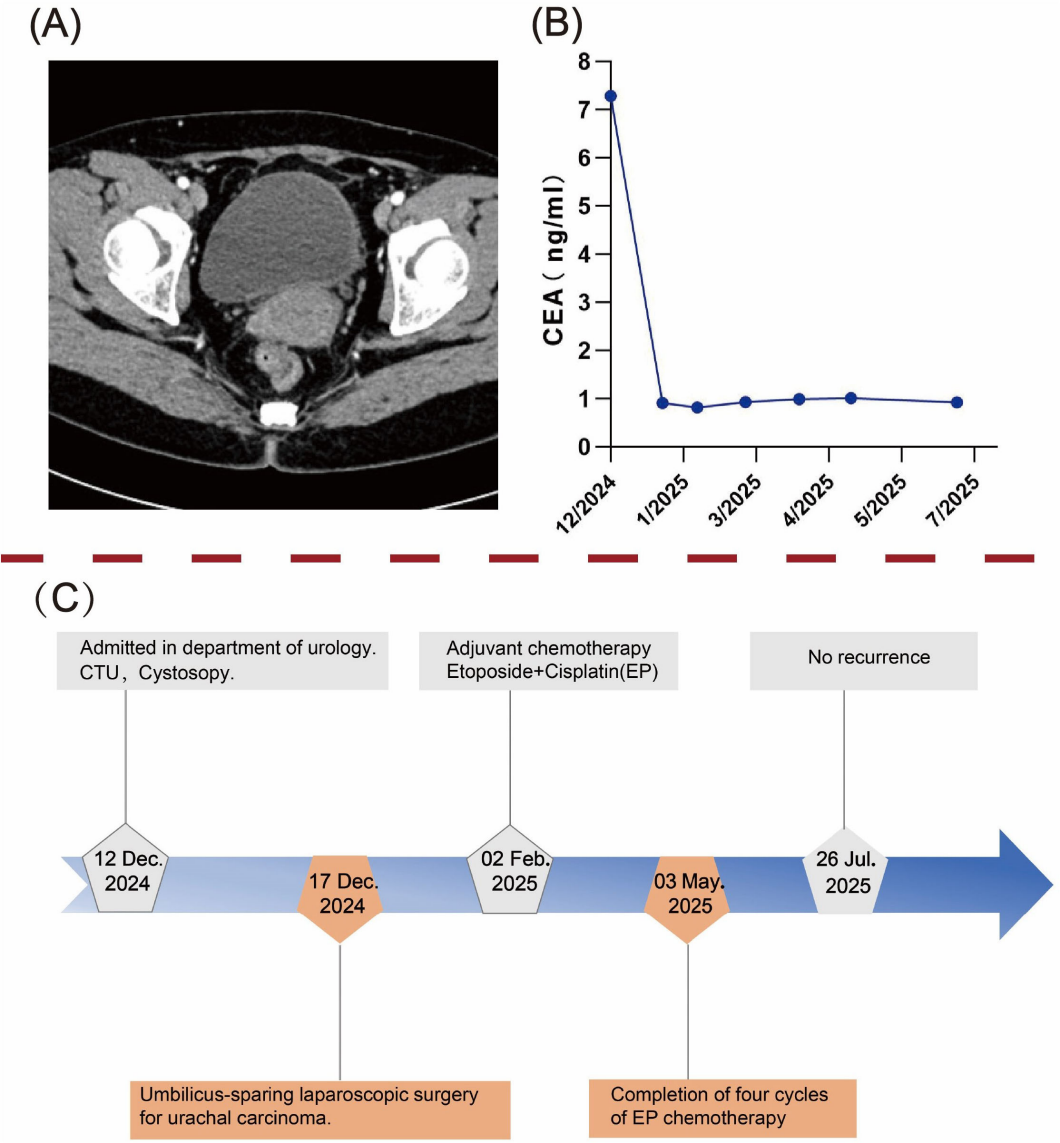
Postoperative follow-up and diagnostic-therapeutic timeline. **(A)** Abdominal computed tomography (CT) scan at 8 months postoperatively showed no evidence of tumor recurrence. **(B)** Dynamic trend of carcinoembryonic antigen (CEA) levels during follow-up. **(C)** A timeline flowchart outlining the diagnosis and treatment process.

Given the high malignancy and aggressiveness of urachal neuroendocrine carcinoma (NEC) (9), the patient underwent four cycles of adjuvant chemotherapy with etoposide plus cisplatin (EP) after multidisciplinary oncological evaluation. No ≥Grade 3 treatment-related adverse events were recorded (CTCAE v5.0), and overall tolerability was good. After umbilicus-sparing extended partial cystectomy and adjuvant chemotherapy, the patient reported good overall quality of life without irritative voiding symptoms or incontinence and expressed satisfaction with the treatment. The patient underwent follow-up evaluations every three months, including tumor markers and whole-abdominal CT scans, and has remained disease-free to date. A timeline summarizing the diagnostic and therapeutic process is illustrated in **Figure 4**.

## 3 Case-based review of urachal neuroendocrine carcinoma

### 3.1 Previously reported urachal NEC cases

The literature search was conducted in PubMed using the keywords “urachal neuroendocrine carcinoma,” “urachal NEC,” “urachal small cell carcinoma,” and “urachal carcinoma.” Six relevant publications were identified, reporting a total of nine patients with urachal neuroendocrine carcinoma (NEC) (**Table 1**) (5, 9–13). Urachal NEC is an exceptionally rare and highly aggressive tumor of urachal origin, first formally described in the literature by Hom et al. in 1990. Among the nine patients, six were male and three were female; the mean age at presentation was 52.4 years, and the mean tumor diameter was 4.6 cm. Most tumors were located at the bladder dome or along the median umbilical ligament, consistent with the embryologic course of the urachus. Gross hematuria was the most common presenting symptom (8/9), with a minority reporting dysuria, flank pain, or infection. Histologically, seven of the nine cases were mixed tumors comprising NEC with an adenocarcinoma component, including six small-cell NECs (SCNEC) and one large-cell NEC (LCNEC); one case was NEC with a urothelial carcinoma component, and the remaining case was pure small-cell NEC. In the six patients with SCNEC combined with adenocarcinoma, the NEC component was predominantly positive for Chromogranin A (CgA), Synaptophysin (Syn), and INSM1. Regarding management, all nine patients underwent initial local therapy: seven received partial cystectomy, one underwent radical cystectomy, and one was treated with transurethral resection of bladder tumor (TURBT). For adjuvant therapy, five patients received platinum-based chemotherapy, and four of these also underwent postoperative radiotherapy. Despite multimodal treatment in some cases—including definitive surgery combined with adjuvant chemoradiation—long-term survival remained suboptimal. Of the eight cases with available follow-up, seven developed distant metastases, most commonly to the lungs, liver, and lymph nodes, and the majority of patients died within 12–24 months of diagnosis, underscoring the highly malignant nature and early metastatic propensity of this disease.

**Table 1:** Summary of previously reported cases of urachal NEC.

Cases	Case 1	Case 2	Case 3	Case 4	Case 5	Case 6	Case 7	Case 8	Case 9
Age (y)	33	23	25	34	56	64	53	31	76
Sex	Male	Male	Female	Male	Female	Male	Male	Female	Female
Presenting symptoms	Hematuria	Hematuria	Dysuria and hematuria	Hematuria	Hematuria	Hematuria	Dysuria and low back pain	Hematuria	Hematuria
Tumor size (cm)	4	4.8	2.5	6	7	2.5	6	3.5	NA
Tumor location	Dome	Dome	Dome	Dome	Dome	Dome	Dome	Dome	Dome
NEC type	SCNEC	SCNEC	LCNEC	SCNEC	SCNEC	SCNEC	SCNEC	SCNEC	SCNEC
NE marker expression	Syn, Chr, CD56	Syn, Chr	Syn, Chr	Chr,NES	Syn	Syn	Chr, NSE	NSE	NA
Other carcinoma	Adenocarcinoma	Adenocarcinoma	Adenocarcinoma	Adenocarcinoma	CIS	NA	Adenocarcinoma	Adenocarcinoma	Adenocarcinoma
Sheldon stage	IV	IIIA	IVA	IVA	IV	III	III	NA	III
Recurrence/metastasis	Lung, liver, lymph nodes, vertebrae	Lungs, pleura, and pelvic and lymph nodes	Brain, lungs, liver, lymph nodes	Liver, spine, lymph nodes, local recurrence	Lungs, spine, mediastinum, and lymph nodes	Lumbar spine	NA	Liver, bones, and pelvis	NA
Treatment	TURBT, Chemo, Rad	PC, PLND, and Chem	PC, PLND, Chem,and Rad	PC, PLND, Chem,and Rad	PC and PLND	CP, PLND, Chem, and Rad	PC and PLND	PC and PLND	PC
Follow-up outcome	Died at 5 mo	NA	Died at 31 mo	Died at 10 mo	Died at 18 mo	Died at 24 mo	NA	Died at 6 mo	Alive at 72 mo
Reference	Obiedat et al., 2024[9]	Wang et al., 2017[13]	Wang et al., 2017[13]	Wang et al., 2017[13]	Paner et al., 2012[5]	Paner et al., 2012[5]	Munichor et al., 1995[12]	Hom et al., 1990[10]	Johnson et al., 1985[11]

CIS, urothelial carcinoma in situ; CP, radical cystoprostatectomy; NA, information not available; PC, partial cystectomy; PLND, pelvic lymph node dissection; Chem, chemotherapy; Rad, Radiotherapy; SCNEC, Small Cell Neuroendocrine Carcinoma; LCNEC, Large Cell Neuroendocrine Carcinoma.

Currently, no standardized treatment consensus exists for urachal NEC. Clinical decisions are generally guided by therapeutic approaches established for NECs of other urologic origins, such as bladder and prostate NEC, emphasizing multidisciplinary evaluation and aggressive systemic therapy. Due to its extreme rarity, available literature is largely limited to individual case reports, and treatment regimens lack support from prospective clinical studies, precluding the establishment of evidence-based strategies. Overall, urachal NEC exhibits highly aggressive biological behavior and poor long-term survival even after surgical and adjuvant therapies. Further accumulation of cases and multicenter studies are urgently needed to standardize and optimize its management.

### 3.2 Embryological and anatomical basis of urachal carcinoma development

The urachus is a midline embryonic remnant derived from the allantois that connects the umbilicus to the bladder dome. Under physiological conditions, it typically undergoes complete obliteration before birth, forming the median umbilical ligament (6, 9, 14). The urachus is a vestigial structure, and incomplete involution of its canal may lead to persistence into adulthood. Autopsy studies suggest that approximately one-third of adults exhibit partial patency of the urachus, which can give rise to congenital anomalies—including cysts, fistulas, or diverticula—or, more rarely, malignant neoplasms (15, 16). Anatomically, the urachus is composed of three histological layers: an inner epithelial lining, a middle connective tissue layer, and an outer smooth muscle coat (17). Primary UrC is an exceedingly rare malignancy, representing less than 1% of all bladder cancers (1, 2). UrC was first identified during autopsy by Hue and Jacquin (18) in 1863, with its pathological features further characterized by Cullen (19) in 1916 and its clinical classification refined by Begg (20) in 1930. These foundational observations paved the way for subsequent exploration into the pathogenesis and clinical management of UrC. According to previous reports, UrC most commonly originates at the bladder dome, the anatomical junction of the urachus and bladder, where chronic irritation and epithelial metaplasia may contribute to tumorigenesis (21–23). UrC may arise from any histological layer of the urachal wall. Adenocarcinoma and urothelial carcinoma most often derive from the epithelial lining, whereas malignancies of mesenchymal or muscular origin include sarcomas, leiomyosarcomas, and the exceptionally rare neuroendocrine carcinoma (NEC) (5, 24, 25).

### 3.3 Clinical manifestations and diagnosis of urachal carcinoma

UrC typically presents with no obvious symptoms during its early stages and often manifests clinically only at advanced stages of disease progression, owing to its inherent tendency for early local invasion and distant metastasis (23, 26). Gross hematuria is the most common clinical presentation, observed in approximately 90% of cases, and is generally attributable to tumor invasion into the bladder (6, 8, 15). Because its early symptoms closely resemble those of primary bladder cancer, UrC is frequently misdiagnosed. However, one distinguishing feature is the presence of mucinous material in the urine, which is more commonly seen in patients with UrC (27). Other frequently reported symptoms include a palpable suprapubic mass, lower abdominal pain, and dysuria (15, 16, 28). The diagnostic criteria for UrC were first proposed by Sheldon et al. (29) in 1984, and subsequently refined by Gopalan et al. (30). These criteria include the following (1): the tumor is located at the dome or anterior wall of the bladder (2); the epicenter of the lesion lies within the bladder wall (3); there is no widespread cystitis glandularis or cystitis cystica beyond the dome or anterior wall; and (4) no evidence exists of a primary tumor elsewhere in the body.

Following the definition of tumor location and exclusion criteria, staging evaluation plays a crucial role in guiding treatment strategies and assessing prognosis for UrC. In 1984, Sheldon et al. (29) proposed a classical staging system that classified UrC into eight substages (I–IVC), reflecting the disease continuum from mucosal confinement to regional lymphatic spread and distant metastasis. However, due to its complexity, the Sheldon system has limited applicability in clinical practice. In 2006, Ashley et al. (23) introduced the more simplified Mayo staging system, which categorizes UrC into four stages: Stage I (confined to the urachal mucosa), Stage II (invasion into the bladder), Stage III (involvement of surrounding soft tissues or lymph nodes), and Stage IV (presence of distant metastasis). This system has been widely adopted in clinical research and practice and demonstrates favorable prognostic predictive value. In the same year, Pinthus et al. (31) proposed the Ontario staging system, which adopts a TNM-like classification (T1–T4) that emphasizes the depth of invasion. To date, multiple retrospective analyses have confirmed the Mayo staging system as the most widely utilized and prognostically informative staging tool for UrC (30, 32).

The principal differential diagnoses of urachal NEC include primary bladder NEC, secondary metastatic NEC, and NEC originating from a bladder diverticulum (5, 9, 13). Imaging examinations play a critical role in the further evaluation and staging of UrC (16, 33). Ultrasound typically reveals a midline, heterogeneous mass with irregular margins located above the bladder dome, which can serve as an initial clue for the suspicion of UrC (6). In contrast, computed tomography (CT) and magnetic resonance imaging (MRI) offer higher spatial resolution and are more suitable for delineating tumor extent, assessing local invasion, detecting lymph node metastasis, and identifying distant lesions (34, 35). UrC demonstrates characteristic radiologic features, typically presenting as a mixed cystic-solid mass originating from the bladder dome, often accompanied by punctate or peripheral calcifications (36, 37). Studies have shown that 32%–46% of UrC cases exhibit typical calcifications on CT imaging, which are considered one of the relatively specific radiological features of UrC (38, 39). Additionally, a retrospective study by Das et al. reported that MRI-based Mayo staging demonstrated up to 90% concordance with postoperative pathological staging, particularly in assessing whether the tumor invades beyond the bladder dome (40). Despite the crucial role of imaging in assessment, UrC often exhibits overlapping features with urothelial carcinoma; therefore, cystoscopy and histopathological biopsy remain the gold standards for definitive diagnosis. Cystoscopy enables direct visualization and precise localization of the lesion in most patients, providing essential diagnostic guidance (15, 23, 41, 42).

Serum tumor markers associated with UrC primarily include cancer antigen 125 (CA-125), cancer antigen 19-9 (CA19-9), and carcinoembryonic antigen (CEA), among which CEA is considered the most sensitive serological indicator (32, 43, 44). Several studies have reported that approximately 55%–60% of patients present with elevated preoperative CEA levels, which often decline significantly following surgery and chemotherapy. This suggests that CEA may serve not only as an adjunctive diagnostic marker but also as a valuable tool for postoperative surveillance, therapeutic response assessment, and prognostic evaluation (9, 16). In the present case, the patient’s preoperative CEA level was 7.28 ng/mL, markedly exceeding the upper normal limit, and subsequently decreased to 0.82 ng/mL after surgery, indicating a strong correlation between CEA expression and tumor burden. These findings further support the potential utility of CEA as a reliable biomarker for monitoring treatment response and prognostic evaluation in UrC.

### 3.4 Histopathological and immunohistochemical features of urachal NEC

Neuroendocrine carcinoma (NEC) is a highly aggressive malignant tumor originating from neuroendocrine cells and is characterized by a strong tendency for early metastasis (5, 45). According to the current classification system, NEC is categorized into four subtypes: carcinoid tumor, atypical carcinoid tumor, small cell neuroendocrine carcinoma (SCNEC), and large cell neuroendocrine carcinoma (LCNEC). Among them, SCNEC and LCNEC exhibit the lowest degree of differentiation and the highest level of malignancy, with a strong propensity for recurrence and distant metastasis, resulting in an extremely poor prognosis (46–48).

Among reported cases of urachal neuroendocrine carcinoma (NEC), small cell neuroendocrine carcinoma (SCNEC) is the most frequently observed subtype. Histopathological examination remains the gold standard for confirming SCNEC (49, 50). According to the World Health Organization (WHO) histological classification, SCNEC can be divided into oat cell, intermediate, and mixed types (51). Under light and electron microscopy, tumor cells appear as sheets or nests of small round cells with hyperchromatic nuclei, scant cytoplasm, inconspicuous nucleoli, frequent mitotic figures, and abundant dense-core neurosecretory granules, reflecting high proliferative activity (51–54). Immunohistochemical staining plays a critical role in the diagnosis of NEC (45, 55). Neuroendocrine markers including synaptophysin (Syn), chromogranin A (CgA), and insulinoma-associated protein 1 (INSM1) are widely used in the diagnosis of genitourinary NEC (56, 57). Syn is a widely expressed synaptic vesicle membrane protein, while INSM1 is a neuroendocrine-specific transcription factor with high sensitivity in both well-differentiated and poorly differentiated tumors (58–60). In contrast, CgA may be negative in high-grade NECs such as SCNEC (61). The Ki-67 proliferation index is a key biomarker for assessing tumor biological behavior. In high-grade NECs, it often exceeds 80%, indicating a high proliferation rate, aggressive nature, and poor prognosis (62–64). Additionally, aberrant expression of P53 (either strong overexpression of mutant type or complete loss) and loss of retinoblastoma protein (Rb) expression are commonly observed in SCNEC, indicating molecular dedifferentiation and serving as references for differential diagnosis and prognostication (65–67). Moreover, cytokeratin 20 (CK20) is expressed in nearly 100% of urachal adenocarcinomas and is frequently accompanied by CDX2 positivity, serving as a crucial immunophenotypic marker for distinguishing enteric from non-enteric adenocarcinomas and clarifying tumor origin (30, 36, 68). In the present case, immunohistochemistry revealed Ki-67 (+, 80%), Syn (+), CgA (+), INSM1 (+), Rb (−), P53 (mutant overexpression), CK20 (+), and CDX2 (+). These findings indicate a high-grade NEC. Based on the microscopic features of small cell morphology and approximately 20% adenocarcinoma component, the final diagnosis was mixed-type urachal SCNEC.

### 3.5 Multimodal management and prognostic assessment of urachal carcinoma

Surgical resection remains the cornerstone of UrC treatment, although no standardized surgical guidelines have been universally established to date (16, 37). Common surgical strategies include en bloc resection of the urachus and umbilicus, radical or partial cystectomy, and bilateral pelvic lymph node dissection (8, 9, 37). Current evidence suggests that partial cystectomy offers oncologic outcomes comparable to those of radical cystectomy, with the added benefits of bladder preservation, improved postoperative quality of life, and fewer complications. It is thus considered the preferred approach, especially for tumors confined to the bladder dome (41, 69). Notably, complete tumor resection with negative surgical margins is a critical determinant of long-term survival (17, 34, 36). Gelli et al. (36) reported that prognosis in UrC is closely associated with pathological stage, margin status, lymphovascular invasion, and whether the umbilicus was resected. Similarly, Harry et al. (2) emphasized that local tumor stage and surgical margin status are the most critical prognostic factors for patient survival. Multiple studies have consistently demonstrated that achieving negative margins through complete resection significantly improves survival outcomes. The prognostic benefit of pelvic lymph node dissection remains controversial. Some studies suggest that bilateral pelvic lymphadenectomy does not significantly improve overall survival and is associated with higher postoperative complication rates, with a nodal positivity rate of only 17% (34). Despite advances in surgical techniques, postoperative recurrence remains frequent, occurring in approximately 20%–38% of patients, and metastatic UrC carries a particularly poor prognosis (37, 41). Common sites of recurrence include the pelvis, bladder, lungs, and lymph nodes (17). Among NEC subtypes, the prognosis is even worse due to their high proliferative activity and aggressive biological behavior (70).

In this context, neoadjuvant or adjuvant therapies are considered potentially beneficial in improving long-term survival in patients with UrC. However, due to the rarity of UrC, no standardized chemotherapy regimen has been established to date (71). Among current regimens, the combination of 5-fluorouracil and cisplatin (5-FU + cisplatin) is the most commonly used and has demonstrated relatively high response rates. However, it is primarily applied in adenocarcinoma-type UrC, and its efficacy in non-adenocarcinoma subtypes such as NEC remains unclear (6, 16, 37, 72). For high-grade subtypes such as SCNEC, there is currently no universally accepted chemotherapy protocol. Treatment strategies for SCNEC are often extrapolated from those for small cell lung cancer and genitourinary small cell carcinoma, with the etoposide plus cisplatin (EP) regimen widely adopted as a first-line therapy and showing modest efficacy (73–75). According to consensus guidelines for genitourinary small cell carcinoma, 4 to 6 cycles of the EP regimen are recommended (76). Radiotherapy is not routinely employed in the treatment of UrC, largely due to its low radiosensitivity (16, 26, 36). Although Mertens et al. (16) explored neoadjuvant radiotherapy combined with intraoperative brachytherapy to improve margin control, this approach has not been adopted in current clinical guidelines. In certain inoperable or metastatic UrC cases, chemoradiotherapy may provide local control or survival benefits; however, robust evidence from systematic studies is lacking (77, 78). Therefore, radiotherapy should be considered a component of individualized or palliative care rather than a standard treatment modality.

In recent years, immune checkpoint inhibitors (ICIs) have made significant strides in the treatment of urologic malignancies. However, the application of ICIs in UrC remains in its infancy, with no prospective clinical trials currently available to validate their efficacy (16, 17) Case reports have demonstrated clinical remission in some patients with recurrent or metastatic UrC treated with PD-1/PD-L1 inhibitors such as pembrolizumab and atezolizumab (71, 79, 80). UrC is most commonly composed of enteric-type adenocarcinoma or exhibits neuroendocrine differentiation, both of which differ substantially from the immune microenvironment of typical urothelial carcinoma (81, 82). In particular, SCNEC is generally characterized by low PD-L1 expression, low tumor mutational burden (TMB), and microsatellite stability (MSS), classifying it as an immunologically “cold” tumor with limited responsiveness to ICIs (83–85). It is worth noting that in cases of mixed histology involving adenocarcinoma components, ICIs may be considered as an exploratory treatment option following failure of standard therapy—especially in tumors exhibiting high PD-L1 expression, elevated TMB, or microsatellite instability-high (MSI-H) status (86). To date, no international guidelines have incorporated immunotherapy into the standard management of UrC. Therefore, its clinical use should be based on individualized assessment supported by biomarker screening and comprehensive evaluation of the patient’s condition.

### 3.6 Current challenges and future perspectives

Urachal NEC is an exceedingly rare and highly aggressive solid tumor, for which robust evidence-based guidelines for diagnosis and treatment remain lacking. Most published data are derived from isolated case reports or small retrospective series, with a notable absence of large-scale prospective studies or clinical trials (36, 87). From a diagnostic standpoint, although imaging, histopathology, and immunohistochemistry can assist in diagnosis, the early clinical manifestations of urachal NEC are often non-specific. Furthermore, its histological and immunophenotypic features may overlap with other urachal tumor subtypes, making early identification particularly challenging (9). Surgical resection remains the mainstay of treatment (16, 37). However, in cases with distant metastases or high-grade histological components, surgery alone may be insufficient for long-term disease control (70). Existing adjuvant chemotherapy regimens are largely extrapolated from treatment paradigms for small cell lung carcinoma or small cell carcinoma of the urinary tract, yet their efficacy in urachal NEC remains unproven due to the lack of systematic validation (73–75). In terms of immunotherapy, although immune checkpoint inhibitors (ICIs) have demonstrated promise across various urologic malignancies, urachal NEC typically exhibits low PD-L1 expression, low tumor mutational burden (TMB), and microsatellite stability (MSS)—characteristics of an immunologically “cold” tumor, which may limit responsiveness to ICIs (83–85, 88).

Future research, in light of current data limitations, may proceed along the following directions: First, establishing multicenter collaborative case registries is essential to enhance understanding of the clinical heterogeneity and prognostic factors of urachal NEC. Second, comprehensive molecular profiling studies should be conducted to identify potential biomarkers that could inform targeted or immunotherapeutic strategies. Third, the development of prospective clinical trials is needed to systematically evaluate the efficacy of adjuvant chemotherapy, radiotherapy, and immunotherapy, either alone or in combination. The integration of precision medicine and multidisciplinary approaches holds promise for optimizing disease management and ultimately improving patient survival and quality of life.

## 4 Discussion

Urachal neuroendocrine carcinoma (NEC) is an exceptionally rare malignancy, with only nine cases clearly documented in the global English-language literature to date. Review of the nine reported cases reveals that urachal NEC typically presents as high-grade, small cell morphology, with a markedly elevated Ki-67 index and positive immunohistochemical staining for Synaptophysin (Syn), Chromogranin A (CgA), and INSM1. Some cases also exhibit adenocarcinoma or signet ring cell components, indicating significant histological and molecular heterogeneity. Existing literature suggests that urachal NEC is characterized by high biological aggressiveness and poor prognosis, with early postoperative recurrence or distant metastasis being common. Due to its extremely low incidence, no standardized diagnostic or therapeutic guidelines have been established. In terms of treatment, most reported cases have undergone umbilical resection combined with radical cystectomy (RC), followed by multiple cycles of platinum-based adjuvant chemotherapy. Nevertheless, overall survival remains limited and the postoperative recurrence rate is high. In select cases with localized disease, partial cystectomy combined with adjuvant chemotherapy has yielded favorable outcomes, suggesting that function-preserving surgery may be a viable option when the tumor is well-demarcated and anatomically confined. This report presents the tenth documented case of urachal NEC, in which the tumor was confined to the bladder dome without evidence of distant metastasis at diagnosis. The patient underwent umbilicus-sparing total urachal resection combined with extended partial cystectomy, followed by adjuvant chemotherapy with an etoposide and cisplatin (EP) regimen. The pathological characteristics, immunophenotype, and treatment strategy of this case were largely consistent with previous reports. Notably, the individualized surgical approach provides a potential reference for function-preserving treatment in comparable cases.

In summary, urachal NEC is an exceptionally rare and highly aggressive malignancy, for which no standardized diagnostic or therapeutic guidelines currently exist. Surgical resection remains the primary treatment modality, with emphasis on complete tumor excision and negative surgical margins. Adjuvant chemotherapy is often guided by treatment protocols established for small cell carcinomas of the lung or urinary tract. Immunotherapy remains investigational and should be considered based on molecular profiling and biomarker selection. This report presents a case of mixed histology urachal NEC and, in conjunction with a review of nine previously published cases, systematically summarizes the clinical features, diagnostic and therapeutic considerations, and prognostic patterns of this rare entity. It provides practical insight and literature-based evidence for individualized treatment strategies in urachal NEC, and may inform future clinical decision-making and research directions.

## Data Availability

The raw data supporting the conclusions of this article will be made available by the authors, without undue reservation.
